# Voxel-Based Lesion Analysis of Ideomotor Apraxia

**DOI:** 10.3390/brainsci14090853

**Published:** 2024-08-24

**Authors:** Giovanna Oliveira Santos, Analía L. Arévalo, Timothy J. Herron, Brian C. Curran, Guilherme Lepski, Nina F. Dronkers, Juliana V. Baldo

**Affiliations:** 1 Department of Experimental Surgery, Medical School, University of São Paulo, Sao Paulo 05021-001, Brazil; analia.l.arevalo@gmail.com (A.L.A.); lepski@gmail.com (G.L.); 2 Research Service, VA Northern California Health Care System, Martinez, CA 94553, USA; timothy.herron2@va.gov (T.J.H.); brian.curran@va.gov (B.C.C.); juliana.baldo@va.gov (J.V.B.); 3 Department of Neurosurgery, Eberhard Karls University, 72074 Tübingen, Germany; 4 Departments of Neurology and Psychiatry, Medical School, University of São Paulo, Sao Paulo 05021-001, Brazil; 5 Department of Psychology, University of California, Berkeley, Berkeley, CA 94720, USA; dronkers@berkeley.edu; 6 Center for Mind and Brain, University of California, Davis, Davis, CA 95616, USA

**Keywords:** chronic stroke, left hemisphere lesions, lesion–symptom mapping, praxis, western aphasia battery

## Abstract

Ideomotor apraxia is a cognitive disorder most often resulting from acquired brain lesions (i.e., strokes or tumors). Neuroimaging and lesion studies have implicated several brain regions in praxis and apraxia, but most studies have described (sub)acute patients. This study aimed to extend previous research by analyzing data from 115 left hemisphere chronic stroke patients using the praxis subtest of the Western Aphasia Battery, which is divided into four action types: facial, upper limb, complex, and instrumental. Lesion–symptom mapping was used to identify brain regions most critically associated with difficulties in each of the four subtests. Complex and instrumental action deficits were associated with left precentral, postcentral, and superior parietal gyri (Brodmann areas 2, 3, 4, 5, and 6), while the facial and upper limb action deficits maps were restricted to left inferior, middle, and medial temporal gyri (Brodmann areas 20, 21, 22, and 48). We discuss ideas about neuroplasticity and cortical reorganization in chronic stroke and how different methodologies can reveal different aspects of lesion and recovery networks in apraxia.

## 1. Introduction

Ideomotor apraxia is a cognitive disorder that most often results from acquired brain lesions, such as strokes or tumors [1,2,3,4,5]. Ideomotor apraxia reflects impairment in an individual’s ability to understand, imitate, pantomime, or perform an action that is presented visually or verbally [6], and may involve different body parts, such as the face (eyes, mouth) or limbs (arms, hands, fingers) [4]. Ideomotor apraxia is most commonly understood as a skilled movement deficit that cannot be explained by motor or sensory impairments, nor by a lack of language comprehension.

The first description of the neural basis of apraxia was postulated by Hugo Liepmann [7,8,9] over 100 years ago. While he suggested a central role for extrastriate visual areas in ideational apraxia, he believed ideomotor apraxia involved damage to the left parietal cortex, where movement ideas were transformed into innervatory engrams, which were then implemented within sensorimotor areas bilaterally. The last few decades have seen the advent of neuroimaging methods, which have also paved the way for lesion–symptom mapping techniques [10,11], tools which have been invaluable in advancing our understanding of the neural underpinnings of apraxia, among other disorders, (e.g., [12,13]). Most tests of apraxia have focused on assessing the processes underlying pantomime versus imitation, the use of different body parts, and different types of actions (e.g., transitive—when the movement involves an object, like hammering a nail—vs. intransitive—no object, like saluting) [13,14].

According to the Two Action Systems model (2AS) [15,16], the brain houses two anatomically separate action systems that are highly interactive: (1) the “ventral stream”, which is left lateralized and stores the core aspects of a gesture, making it recognizable in different contexts and linking the object to its functional use; and (2) the “dorsal stream”, which functions bilaterally and processes the structure of an object and spatiomotor information about one’s own body. In this model, the dorsal stream is further subdivided into a “dorso-dorsal stream”, specialized in online control of grasping, planning, and executing gestures related to object structure, and a “ventro-dorsal stream”, specialized in processing action recognition and skilled action.

Hoeren et al. [17] investigated the role of the dorso-dorsal, ventro-dorsal, and ventral streams in imitation and pantomime. In their study, 96 patients with acute left hemisphere stroke were asked to imitate meaningless hand and finger gestures as well as pantomime tool use. Voxel-based Lesion Symptom Mapping (VLSM) [10] analyses showed that imitation was largely associated with the dorso-dorsal stream (mainly the posterior inferior parietal sulcus and the superior parietal lobe), areas associated with the maintenance of body schema, online spatial relations between body parts and environment, and construction of high-level representations of perceived gestures; on the other hand, pantomime was more closely associated with ventro-dorsal regions associated with stored action representations (anterior inferior parietal lobe and posterior middle temporal gyrus). The authors suggest that the ventral pathway (containing the insula and the subinsular white matter) may facilitate top-down activation of movement engrams, selection of relevant movement features, and integration of motor programs with semantic knowledge.

In another VLSM study of pantomime in 150 patients with acute stroke or recently removed tumors, Manuel et al. [18] reported that spatial configuration (SC) and body part-as-object (BPO) errors were associated with damage to left inferior frontal regions. Furthermore, left parietal, frontal, and temporal areas dissociated according to error type and lesion etiology: (I) while SC errors were more associated with lesions in left inferior parietal areas, BPO errors were more associated with lesions in the inferior to superior frontal gyrus; (II) in stroke patients, spatial configuration errors were less associated with parietal areas and more associated with temporal areas when compared with tumor patients.

In Schmidt et al. [19], 91 subacute left hemisphere stroke patients performed several tasks, including gesture imitation, pantomime, and actual object use, with arm/hand-, finger-, and face-related actions. Using VLSM and a Principal Component Analysis (PCA), imitation of arm/hand gestures was associated with the supramarginal gyrus, the angular gyrus, the precentral gyrus, and the posterior superior temporal gyrus. Furthermore, imitation of finger gestures alongside buccofacial pantomime and actual object use were associated with the angular gyrus and the inferior parietal sulcus. Finally, buccofacial imitation alone was associated with basal ganglia, white matter tracts, other subcortical structures, the posterior superior temporal gyrus, and the postcentral gyrus, which the authors suggest indicates a relationship between the kinematic component of actions and the inferior parietal lobe and other precentral regions.

While the studies above focused on acute or subacute patients, fewer studies using the most recent lesion–symptom techniques have investigated lesion correlates of ideomotor apraxia in chronic patients. In Rounis et al. [20], 41 chronic stroke patients completed a large test battery to assess multiple aspects of praxis (gesture recognition, pantomime, single and multi-object use, meaningless gesture imitation, and complex figure copy). Using a voxel-based correlational methodology [21], the authors identified a role for the ventro-dorsal stream in semantic control of actions and tool gestures and a role for the dorso-dorsal stream in sequencing actions to achieve a specific goal.

The extent and nature of the structural and functional differences between (sub)acute and chronic stroke populations is an area of interest to many researchers. Basso et al. [22] conducted a retrospective study following the recovery pattern of 44 ideomotor apraxia patients with a single left hemisphere lesion across three time points: first, within 1–3 months post-onset; second, at least 4 months after the first; and third, at least 6 months after the second (ensuring a minimum of 18 months post onset). The authors found that patients with frontal lesions exhibited a more severe degree of ideomotor apraxia at all examination points compared with those with parietal lesions, but the degree of recovery was not significantly different between groups. Most patients showed significant recovery from ideomotor apraxia within the first few months following the lesion, after which progress typically hit a plateau; however, some patients continued to improve, while others experienced a decline, highlighting the role of individual variability in recovery.

Many authors agree that key skills can and do rehabilitate over time, with several important results emerging in recent studies. For language, for example, recovery may occur over long periods of time [23]; however, some recent techniques such as repetitive transcranial magnetic stimulation (rTMS), which may assist language recovery in the acute phase, may not be as effective in chronic patients [24], suggesting there are limitations to the plasticity of brains in the chronic phase of recovery. Unlike language skills, apraxia has been found to be more persistent, with most recovery occurring within the first few months after stroke [25]. While imitation and actions with real tool use seem to recover best, gesturing to verbal commands may not recover as well. These findings indicate that the (sub)acute and chronic populations reveal different aspects of apraxia, each exposing a different stage in the process. As we would expect both structure and function to be more stable in the chronic phase, findings with chronic patients can best inform practice with chronic patients. Thus, studying several populations is key to gaining a broader picture of apraxia, both structurally and functionally, at several stages of recovery.

As most studies so far have focused on acute patients, in the current study, we aimed to extend previous investigations of ideomotor apraxia by analyzing data from 115 left hemisphere stroke patients on the praxis subtest of the Western Aphasia Battery (WAB) [26,27]. The WAB praxis subtest evaluates four distinct dimensions of praxis: upper limb/hand actions, face/mouth actions, instrumental actions, and complex actions. Unlike previous studies that designed their experiments to test imitation and pantomime independently, the WAB does not allow for this comparison. In the WAB, each item is presented orally, and patients are asked to attempt to pantomime its use. If the patient is not able to correctly perform the pantomime, the examiner demonstrates the action and the patient is given the chance to imitate, for a lesser score. Furthermore, the complex and instrumental subtests add additional demands, such as performing actions associated with objects (transitive) or with sequential movements. However, since transitivity occurs across subtests, it was not analyzed separately here. Nonetheless, the separate upper limb/hand and face/mouth conditions allowed for us to examine performance on actions involving these body parts separately, as few other studies have done [28,29,30]. Thus, our goal was to utilize this large patient sample to identify critical brain regions underlying ideomotor apraxia that extend into the chronic phase of stroke.

## 2. Materials and Methods

### 2.1. Participants

In this retrospective study, we analyzed lesion and behavioral data from 115 participants recruited at a large healthcare system in California. The subjects were part of a larger study in which extensive neuropsychological testing and brain imaging data were collected. All selected participants met the following inclusion and exclusion criteria: normal or corrected to normal vision and hearing; right-handed; native English; and no prior history of psychiatric or neurological disorders. Only patients with a single, identifiable infarct confined to the left hemisphere were included in the analyses (as assessed by a board-certified neurologist from MRI and/or CT scan). All patients signed informed consent forms prior to participation, and this study was approved by the local Institutional Review Board and conducted in accordance with the Helsinki Declaration.

Patients’ mean age was 60.14 years (SD = 11.79, range 31–86). All patients were in the chronic phase of stroke (>3 months post-stroke), with a mean time post-stroke of 45.3 months (SD = 50.29, range 12–271). Mean years of education was 15.10 (SD = 2.31, range 12–20) and mean lesion volume was 114.80 cc (SD = 94.35, range 0.12–450.79 cc), as shown in Table 1.

Patients varied with respect to the degree of language impairment. Aphasia classifications on the WAB included Anomic (*n* = 37), Broca (*n* = 26), Wernicke (*n* = 10), Global (*n* = 3), Conduction (*n* = 6), Transcortical Sensory (*n* = 3), and Within Normal Limits (WNL; *n* = 30).

### 2.2. Behavioral Data

As part of a larger test battery, patients were administered the Western Aphasia Battery (WAB), a standardized speech and language test that also includes several optional cognitive subtests. While it is not a test designed specifically to test apraxia, it does contain a praxis subtest with several subsections, which in a large cohort of patients with imaging data can provide interesting insights into the underpinnings of the praxis network.

The praxis subtest of the WAB consists of 20 items divided into four different subsections: facial actions; upper limb actions; instrumental actions; and complex actions. Patients are asked to execute each of the 20 proposed movements, as follows: (i) facial: “put out your tongue”, “close your eyes”, “whistle”, “sniff a flower”, “blow out a match”; (ii) upper limb: “make a fist”, “salute”, “wave goodbye”, “scratch your head”, “snap your fingers”; (iii) instrumental: “use a comb”, “use a toothbrush”, “use a spoon to eat”, “use a hammer”, “use a key”; (iv) complex: “pretend to drive a car”, “pretend to knock at the door and open it”, “pretend to fold a paper”, “pretend to light a cigarette”, “pretend to play the piano”. If the patient fails to perform the action indicated by each command, he/she is asked to imitate the gesture performed by the examiner, which results in fewer points. Fewer points are also given for performance that is not completely accurate. The highest score possible on the praxis subtest is 60 points.

### 2.3. Lesion Reconstructions and Analyses

Patients were scanned on a 1.5T or 3T Phillips Eclipse MRI scanner for high-resolution T1-weighted structural 3D MRI scans (50 patients’ 2D recons; 49 patients’ 1.5T 3D recons; and 11 patients’ 3.0T 3D recons). Images were acquired with a Spoiled Gradient Recall (SPGR) sequence (TR/TE = 15/4.47 ms, FOV = 240 mm, 256 × 256 imaging matrix, flip angle = 35°, 0.94 × 1.3 × 0.94 mm^3^ voxels, 212 coronal slices). Patients’ lesions were traced directly onto T1 scans using MRIcron software (v1.0.20190902) [31], and a trained expert blind to the patients’ diagnoses and study hypotheses reviewed the reconstructions for accuracy. Scans were then non-linearly transformed into MNI space (152 MNI template) in SPM. Lesion masks were created for each reconstruction so that the presence of the lesion would not distort the SPM normalization procedure (i.e., cost function masking).

Whenever MRI was not possible (5 patients), CT images were acquired on a Siemens Somatom Emotion 16 CT scanner with 3 × 3 × 3 mm imaging and reconstructed onto an 11-slice, standardized template, see [32,33]. The templates were then digitized with in-house software and non-linearly transformed into MNI space [34] using SPM5 running on MATLAB software (R2024a, Mathworks, Natick, MA, USA). For this transformation, slices from the two templates were aligned using 50 control point pairs to match anatomical features on the two templates, and the slices were then aligned using a local weighted mean transformation implemented by the cpselect, cp2tform, and imtransform functions in MATLAB 6.5. These algorithms were then used to warp all the lesion reconstructions from the 11-slice template into MNI space. Reliability has been demonstrated previously using this technique [32,33].

**Figure 1:**
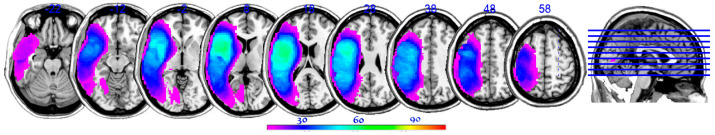
Overlay map of lesions of the 115 patients included in this study. The color bar shows the minimal overlap of five patients per voxel (in violet) up to the maximal overlap of 61 patients (in green).

Lesion–symptom mapping (LSM) software (version 2, 2010) [10,11,35] was used to identify brain regions associated with the 4 different praxis subsections. Both univariate and multivariate LSM maps were generated. For univariate LSM (ULSM), a series of *t*-tests is conducted at every voxel to compare behavioral performance in patients with and without a lesion in that voxel. Only voxels for which at least 5 patients had a lesion were considered in this analysis. Colorized maps of the resultant t or *p* value at each voxel are generated, with hotter colors representing more significant values. To determine the cut-off for a significant cluster size, permutation testing was used based on 1000 iterations and alpha threshold set at 0.05, see [36]. The maps reported are permutation-based Nu = 125 thresholding maps and all analyses were run using the following variables as covariates: lesion size, age, log months post onset, and WAB auditory comprehension score. This last covariate is particularly important to minimize confounding from patients’ difficulty understanding task instructions. The brain regions corresponding to the significant voxel locations were identified utilizing the AAL and Brodmann atlases in MRIcron. Figure 1 shows an overlay of all 115 patients’ lesions, indicating the range of affected brain regions across the left hemisphere.

## 3. Results

### 3.1. Behavioral Results

Demographic and behavioral data for the 115 patients are shown in Table 1, as well as the mean overall praxis score (OPS; maximum possible score of 60), mean Aphasia Quotient (AQ), and Cortical Quotient (CQ). The AQ is a weighted average of all spoken language WAB subscores—namely, spontaneous speech, auditory verbal comprehension, repetition, naming, and word finding—and is the score used to define each patient’s aphasia type, see [24,25]; CQ is a weighted average of all subtest scores, ranging from 0 to 100.

The mean OPS for all 115 participants was 52.63 (SD = 10.57, range 17–60). Mean AQ was 71.67 (SD = 28.25, range 9.1–100) and mean CQ was 74.60 (SD = 24.09, range 17.33–99.95). These scores reveal a wide range of praxis, language, and overall impairment among the patients included in the study.

Although the LSM analyses did not separate patients according to behavioral scores or aphasia classifications, we were interested in knowing how each group fared on each of these measures: as expected, the WNL group had the highest mean OPS (58.96, SD = 1.82), followed by the Anomic group (57.69, SD = 2.15) and the Conduction group (57, SD = 2.58). Patients in the Transcortical Sensory group had a mean OPS of 51 (SD = 6.55), followed by patients with Broca’s aphasia (46.34, SD = 9.83), then Wernicke’s aphasia (36.88, SD = 11.94), and finally patients with Global aphasia (23.33, SD = 10.96).

Table 2 shows the mean, minimum, and maximum scores obtained by participants on each of the four praxis subtests: upper limb, facial, instrumental, and complex (see Section 2.2 for more details). The highest mean scores were obtained for the upper limb subtest (13.63, SD = 2.26, range 5–15), followed by instrumental (13.29, SD = 2.83, range 3–15), facial (13.10, SD = 2.97, range 2–15), and complex (12.68, SD = 3.39, range 0–15).

### 3.2. LSM Results

We conducted LSM analyses on each of the four praxis subtests to assess which regions were critical for performance on each of the tests individually. Data were acquired using permutation-based Nu = 125 thresholding maps. All analyses were run using lesion size, age, log months post onset, and WAB AQ scores as covariates. Table 3 shows the main clusters for each subtest.

**Figure 2:**
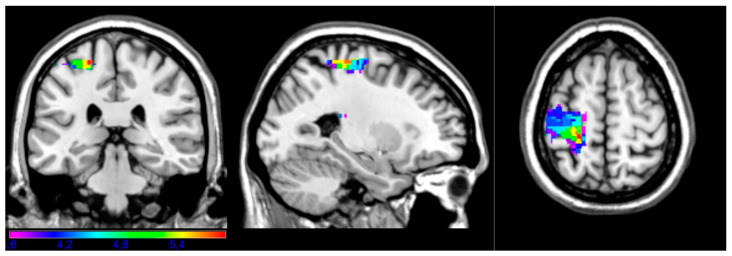
Complex subtest. Map showing the regions significantly associated with impaired performance on the complex task (e.g., “pretend to drive a car”, “pretend to knock at the door”). Areas included BA 2, 3, 4, and 6 (precentral gyrus, postcentral gyrus, superior parietal gyrus) and white matter.

Figure 2 shows the most critical regions associated with performance on the complex subsection (Figure 2). On that subtest, patients are asked to “pretend to drive a car” or “pretend to knock on the door”, which require the sequencing and articulation of different motor acts. Critical areas were observed in the left precentral gyrus, postcentral gyrus, superior parietal gyrus, and anterior corona radiata white matter (BA 2, 3, 4, and 6). The maximal t-value of 5.89 was obtained in the left precentral gyrus (x = −24, y = −30, z = 62). For this analysis, the minimum t-value cut-off was 3.60.

**Figure 3:**
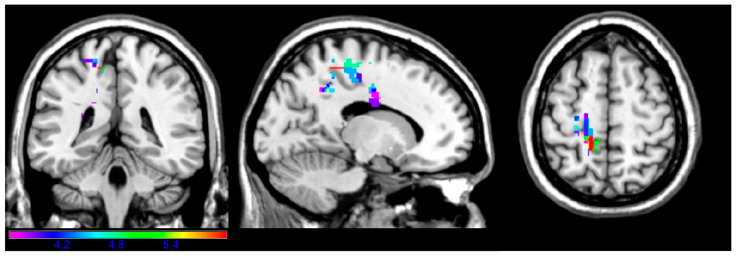
Instrumental subtest. Map showing the regions significantly associated with impaired performance on the instrumental task (e.g., “use a comb”, “use a toothbrush”). Areas included left BA 3, 4, 5, 6, and 11 (precentral and postcentral gyri, inferior frontal orbital cortex) and the precuneus.

Roughly overlapping but fewer areas were associated with performance on the Instrumental subtest (Figure 3), which includes commands such as “use a comb” or “use a toothbrush”. Critical voxels emerged in the left precentral and postcentral gyri (BA 3, 4, 5 and 6); the precuneus, where we measured the maximal t-value of 5.99 (x = −14, y = −44, z = 58); and left inferior frontal orbital cortex (BA 11). For this analysis, the minimal t-value cut-off was 3.61.

**Figure 4:**
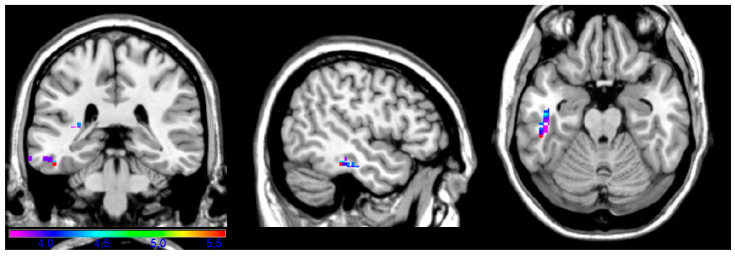
Facial subtest. Map showing the regions significantly associated with impaired performance on the facial task (e.g., “put out your tongue”, “close your eyes”). Areas included BA 20 and 48 (inferior temporal gyrus).

In contrast to the complex and instrumental analyses, the maps for the facial and upper limb subtests yielded considerably fewer critical regions. Furthermore, these regions were in temporal lobe and not in frontal and parietal cortices, as seen for the two previous maps. The facial subtest required patients to perform motor acts related to facial aspects, such as “put out your tongue” or “close your eyes”; critical voxels for that map were in inferior temporal gyrus (BA 20 and 48; Figure 4). The maximal t-value (5.58) in the facial subsection was found in the inferior temporal gyrus (x = −50, y = −34, z = −20). The minimal t-value cut-off used in this analysis was 3.67.

**Figure 5:**
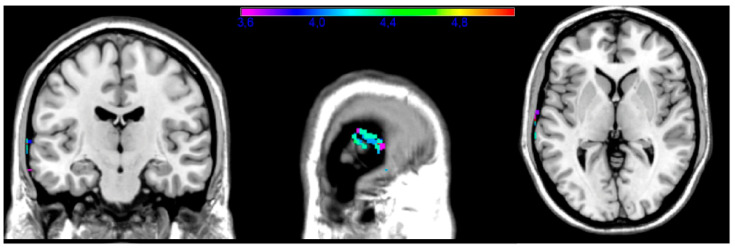
Upper limb subtest. Map showing the regions significantly associated with impaired performance on the upper limb task (e.g., “make a fist”, “salute”). Areas included BA 20, 21, and 22 (inferior and medial temporal gyri).

Commands in the upper limb subtest include “make a fist” and “salute”. Impaired performance on this task was associated with the inferior, medial, and superior temporal gyri (BA 20, 21, and 22; Figure 5). The maximal t-value (5.09) in the upper limb subsection was found in the medial temporal gyrus (x = −72, y = −16, z = 2). In this analysis, the minimal t-value cut-off was 3.57.

## 4. Discussion

Elucidating the neural correlates of ideomotor apraxia allows for a better understanding of which areas are most critically implicated in skilled movement and movement disorders. Tasks that test praxis in healthy and patient populations vary widely and tap a number of different processes including semantic memory, knowledge of tool use, action and gesture recognition, skilled action production, grasp control, motor planning and execution, and ability to manipulate objects based on their properties (i.e., integrating knowledge of structure and function), among others. Thus, depending on the tasks and populations, neuroimaging and lesion studies have implicated a number of different brain regions, including bilateral frontal, parietal, and temporal cortices, as well as underlying white matter networks.

In a comprehensive review, O’Neal et al. [37] highlighted the prominent role of the left hemisphere in processing knowledge of overlearned actions, which is a critical part of praxis. Within the left hemisphere, critical regions associated with praxis in both neurological patients and healthy participants included parietal cortex (recognition and imitation of pantomimed actions), frontal cortex (action representation), and left temporal cortex (knowledge of tool use and semantic memory) [6,18,37].

Our study aimed to extend previous research on the neural basis of ideomotor apraxia by analyzing a large group of left hemisphere stroke patients (*n* = 115) for whom brain lesion and behavioral data on a standard praxis test were available. Our goal was to further investigate different forms of ideomotor apraxia by creating LSM maps of the regions most critically involved in each of the four subsections of the Western Aphasia Battery’s praxis subtests: upper limb, facial, complex, and instrumental actions.

Overall, our findings suggest that ideomotor apraxia in the chronic stage involves frontal (pre- and postcentral gyri), parietal (superior parietal gyrus and precuneus) and temporal (inferior, middle, and superior gyri) regions of the left hemisphere.

In line with a few previous studies, complex and instrumental praxis was associated with left precentral and postcentral gyri as well as the superior parietal gyrus (for complex actions only) [18,38]. Arguably, the instrumental task is simpler than the complex task, which involves additional motor planning of short sequences of events (e.g., “pretend to drive a car”). It is expected that deficits in both of these sets of tasks would be associated with lesions in premotor, sensory, and superior parietal regions, all involved in motor execution, imagery, and planning. Interestingly, Dressing et al. [39] observed that patients with lesions in ventral premotor cortex were more likely to have persistent production errors in pantomime.

Limb and buccofacial apraxia have been found to be at least partially independent neural correlates in some studies [28,29,30], which have described patients with chronic and acute brain lesions with a double dissociation deficit. Patients with limb apraxia, for example, were significantly more impaired performing transitive actions than intransitive ones, which was not observed in patients with buccofacial apraxia [28]. In Raade et al. [28], limb-specific deficits were correlated with frontal cortical and subcortical regions in the left hemisphere, while no specific pattern was found for buccofacial apraxia. Conversely, Scandola et al. [29] observed a significant involvement of the fronto-insular cortex and its subcortical projections in buccofacial apraxia and detected a correlation between frontal and parietal cortical and subcortical networks with limb apraxia.

Alternatively, in our study, the upper limb and facial praxis subtasks were associated with critical regions restricted to the left temporal lobe (inferior temporal lobe for both and middle temporal lobe for the upper limb only). Two previous studies investigated this distinction in subacute patients. In Schmidt et al. [19], imitation of arm/hand movements and buccofacial pantomime involved posterior superior temporal gyrus; but in addition, arm/hand imitation involved pre- and postcentral gyri, the angular gyrus and the supramarginal gyrus, while buccofacial pantomime involved the postcentral gyrus, basal ganglia, and white matter tracts. While Schmidt et al. [19] distinguished between upper limb and buccofacial movements, the two types of stimuli occurred in two different conditions: imitation versus pantomime. In the WAB, pantomime and imitation are scored on a continuum, which does not allow for us to distinguish between these two skills within the scores obtained by the patients included in our study. In Kleineberg et al. [40], deficits in both arm/hand and buccofacial imitation were associated with lesions in supramarginal gyrus and superior temporal gyrus; within the territory of the left middle cerebral artery, impaired imitation of buccofacial gestures was associated with more anterior lesions, while arm/hand imitation deficits were associated with more posterior lesions. Importantly, the patients included in these two studies were all subacute, which may also explain the difference in our findings.

While parietal areas have been the hallmark of apraxia throughout most of the literature thus far, several studies have exposed the specific role of the temporal lobe in apraxia, and our current findings support these data. For example, Tessari et al. [41] performed lesion–symptom mapping in patients with damage to either the left or right hemisphere and found that critical regions in the left hemisphere associated with the imitation of meaningful gestures were superior temporal gyrus and angular gyrus. In Pizzamiglio et al. [42], 387 subacute patients were analyzed with lesion symptom mapping and the results challenged the traditional model. Specifically, the authors found no significant regions in inferior parietal areas in the pantomime tasks; instead, they observed a critical role for superior and medial temporal areas, as well as underlying white matter connections between the temporal cortex and inferior frontal gyrus (uncinate fascicle) as well as the superior longitudinal fasciculus underlying the primary motor cortex. The authors point out that lesion mapping methods are different from methods like fMRI in that they may not reveal how lesions in one area may impact function in another area connected to it. In other words, lesion methods will reveal regions that are most critical for the task at hand to be performed with high accuracy. As such, these are not the only regions thought to be involved in praxis, as an fMRI study of the same patients would surely reveal the involvement of several more regions, including perhaps inferior parietal regions. Importantly, LSM analyses allow for one to analyze scores on a continuous scale. And, since the patients in this study did not perform at floor levels, the maps also reveal that lesions in those critical areas do not lead to completely impaired function on the subtests tested; instead, lesions in those regions predict greater difficulty on those tasks and are thus deemed critical hubs in the profile of chronic apraxia revealed by these analyses conducted with strict correction methods. Finally, the fact that these maps were created with data from a large number of patients in the chronic phase suggest that this may be the profile observed in brains that have had several months to years to reorganize and may thus differ from the patterns observed or expected in acute populations.

In line with this idea, Rosenzopf et al. [43] suggest that heterogeneous topographical results in previous lesion mapping studies might not only result from differences in study design, but also from the general methodological limitations of univariate topographical mapping in uncovering the structural praxis network. They used diffusion tensor imaging and fractional anisotropy mapping to map the human praxis network and found relevant pathological white matter alterations in a densely connected fronto-temporo-parietal network of short and long association fibers, as well as a striking role of middle and superior temporal lobe disconnection, including temporo-temporal short association fibers, suggesting strong involvement of the temporal lobe in the praxis network. Further, the results stressed the importance of subcortical disconnections for the emergence of apractic symptoms. In sum, combined data from different techniques and populations certainly paints a broader picture of apraxia that extends beyond the classic areas discussed thus far.

As mentioned previously, several researchers have highlighted the importance of studying acute and chronic patients separately. In one study, Busby et al. [44] used univariate region-based lesion-symptom mapping to compare acute versus chronic stroke patients with aphasia. The authors found that while several lesion-to-behavior predictions were very similar between groups, these were mostly broad measures like overall aphasia severity, but not more specific deficits. Also, while the acute lesion/perfusion patterns observed among acute patients may capture the critical changes in underlying vascular territories immediately following stroke, studying chronic patients may provide further understanding of recovery patterns as well as reveal additional regions that may suffer further degeneration over time. Thus, collecting data from both populations provides valuable information that can better inform the care of patients at different stages of recovery.

More specific to apraxia, Dressing et al. [39] examined 90 patients both during the acute and chronic phases and observed several key differences between groups in terms of lesion and recovery patterns. While most patients recovered in terms of conceptual errors, meaningless imitation and tool use production deficits persisted over time. Interestingly, persistent production errors in pantomime were associated with more anterior lesions in the ventro-dorsal stream, specifically ventral premotor cortex. In our group of patients, deficits in the complex subtest of the WAB (e.g., “pretend to play the piano”) were also associated with the more anterior precentral gyrus (which was not reported in [20], the other study focusing on chronic patients). In addition, conceptual errors in pantomime were associated with supramarginal and temporal gyrus. Unlike most other studies cited—but see [41,42]—we also report critical area in temporal regions for the facial and upper limb maps. Finally, Dressing et al. [39] report that lesions in angular gyrus and insula were associated with recovery. Indeed, these areas did not come up as critical in our maps of chronic patients.

Some authors have pointed out the importance of testing for anosognosia, which may be present in different patient populations. Indeed, studies have reported that the lesion correlates of anosognosia for apraxia involve left precentral and postcentral gyri, as well as temporal lobe regions also observed in the LSM maps generated in the current analyses [29,45]. Because we did not test for anosognosia as part of the current battery, we cannot correct for this variable in the present analyses. While several tests for anosognosia are available, they are often difficult to administer in populations with language deficits. However, given the cited findings, future studies should look for ways to best include these assessments and correct for this variable whenever possible.

These data were retrospectively analyzed from a larger study of aphasia that focused on identifying the brain basis of speech production and comprehension, and this praxis test was just part of the standard aphasia battery. While there were no originally specific predictions or plans for these data, it was an opportunity to go back, analyze the subtest, and explore the neural correlates of apraxia. To avoid bias, all available patient data for this subtest were included in the current analysis.

One limitation of our study is that the patients only had left hemisphere lesions, and so we were only able to investigate the involvement of that hemisphere in praxis performance. Another limitation of the WAB praxis subtests is that upper limb and facial gestures are contained in some of the items tested in the complex and instrumental subtests, so it was not possible to dissociate the involvement of specific effectors from the more complex tasks. Nevertheless, the upper limb and facial tasks are useful in that they represent simpler gestures involving the effectors without the added complexity of multiple sequences of gesture planning. Thus, the appearance of voxels in these maps suggest those areas may indeed be critical for gestures specifically involving the effectors and are not due to overall task difficulty. More importantly, these areas appear to be critical beyond the stage of dynamic cortical reorganization since chronic patients were studied in the current study. Finally, the WAB is also limited in that instructions for one part of the test are verbal, which may be more difficult for some patient groups than others. As mentioned above, patients are given the chance to imitate gestures performed by the tester for partial points. For our analyses, we corrected for this by including WAB auditory comprehension scores as a covariate. Nonetheless, the ideal test would have more forms of instruction.

## 5. Conclusions

The present study contributes to our understanding of ideomotor apraxia in chronic left hemisphere stroke by providing LSM and behavioral data from a large group of patients on a task that differentiates among different effectors, tools, and levels of complexity. These data deepen our understanding of the lesion and behavioral profiles of chronic ideomotor apraxia, which can provide information on which types of deficits are expected to appear and thus help in the development of more specific therapies. In sum, our results advance our knowledge on how clinical and lesion aspects of ideomotor apraxia can change over time, showing a picture of brain plasticity and reorganization after stroke that may help inform efforts towards rehabilitation.

To further our understanding of praxis and ideomotor apraxia in chronic patients, future studies should apply tasks that test the skills involved in the subtests as separately as possible, utilize tests that bypass verbal-only instructions, and investigate the involvement of the right hemisphere.

## Figures and Tables

**Table 1:** Patient information and praxis scores.

	Mean	SD	Min	Max
Age (years)	60.14	11.79	31	86
Years of education	15.10	2.31	12	20
Lesion volume (cc)	114.80	94.35	0.12	450.79
Months post-onset	45.30	50.29	12	271
Overall praxis score	52.63	10.57	17	60
AQ ^1^	71.67	28.25	9.1	100
CQ ^2^	74.60	24.09	17.33	99.95

^1^ AQ (Aphasia Quotient): weighted average of all spoken language WAB subscores, used to define each patient’s aphasia type. ^2^ CQ (Cortical Quotient): weighted average of all subtest scores, ranging from 0 to 100.

**Table 2:** Mean scores on WAB praxis subtests.

Subtest	Mean	SD	Min	Max
Upper limb	13.63	2.26	5	15
Instrumental	13.29	2.83	3	15
Facial	13.10	2.97	2	15
Complex	12.68	3.39	0	15

**Table 3:** Peak voxel clusters for each of the WAB subtests.

Subtest	Volume	Px	Py	Pz	Min T	Max T
Complex	614	−24	−30	62	3.60	5.89
Instrumental	356	−14	−44	58	3.61	5.99
Facial	88	−50	−34	−20	3.67	5.58
Upper limb	68	−72	−16	2	3.57	5.1

## Data Availability

The data that support the findings of this study are available from the co-author J.V.B, upon reasonable request. The data are not publicly available due to privacy and ethical restrictions.
